# Chloroform—Pro and Con.

**Published:** 1870-07

**Authors:** N. R. Smith


### ARTICLE VII.

### *Chloroform—Pro and Con.*

By Professor N. R. Smith.

Is the question yet settled whether the introduction of anæsthetic agents in surgical practice is, after all, a blessing to mankind, and a safe and useful adjuvant to surgery? I am aware that many will regard the mere suggestion of this question as heretical; but surely the disastrous results from the employment of the powerful agent, chloroform, have been recorded in a sufficient number of instances to justify the query.

The question which I discuss is not whether chloroform may not be so employed as to be a useful agent; but whether, employed as it is and has been, it has been productive of more good than evil.

When we subject to a painful operation a patient whose constitution, and the state of whose nervous system, are favorable to the salutary effects of chloroform, and when, on the administration of that agent, we see what would otherwise be a painful operation accomplished without the infliction of the least suffering, the operator himself exults in the possession of such an agent, and the unprofessional bystanders are filled with astonishment and admiration.

To such an extent have the happy effects of chloroform been extolled by the profession, that it is not surprising that the public generally should be inspired with unqualified confidence in the agent. This confidence is the more readily accepted because the mind is so willing to adopt that which promises so much. When the transfusion of blood was introduced and practiced in Europe, with the promise of restoring youth to age, the public mind was wonderfully excited. Hundreds offered themselves as the subjects of the experiment, and many deaths resulted from the practice. The governments of Europe were obliged to interfere and forbid the practice.

Now I wish it to be understood, *in limine*, that I employ chloroform in surgical practice, and shall continue to employ it. I merely wish to point out many of the evils resulting from its use, with the hope that those evils may be, in a degree, avoided.

Not the the smallest of the evils arising from the use of chloroform results from the overweening confidence of the public in its efficacy and its safety. They are, therefore, unwilling to submit to the most trifling operation without being placed under its influence, when, perhaps, the administration of the anæsthetic is a far more perilous matter than the operation itself.

The profession are well aware that the deaths from the use of chloroform, even in the hands of scientific men, amount to hundreds, and, if all the cases were truthfully reported, probably to thousands. Men have died from its influence who have, on former occasions, breathed it with impunity; many have perished from it who labored under no organic or functional disease which would seem to forbid its use.

But it is not alone by the sudden extinction of life that chloroform is productive of bad results. The secondary effects of it must by no means be overlooked. It is a grave question, which, perhaps can not yet be satisfactorily answered, does chloroform contribute to the successful issue of surgical operations? The great object of the surgeon is to cure his patient. If he can accomplish this without the infliction of pain, so much the better; but the great end is to *cure his patient*, pain or no pain. If the use of an anæsthetic diminish the probability of his recovery, it is undoubtedly to be rejected.

Are surgical operations as happy in their results as they were before chloroform was introduced? Since anæsthetics were introduced, which is some twenty-five years, if I mistake not, I have constantly employed them in very extensive surgical practice, and, although I have not kept such accurate records of my cases as would enable me to make out statistical tables, I think I can conclusively compare the results of operations under chloroform with those performed without it. I therefore confidently say, that the disastrous sequelæ of such operations have been more common when chloroform has been employed. The first disastrous result to which I allude is secondary hemorrhage.

It is well known that both chloroform and the ethers subdue the force of the circulation, especially in the smaller arteries. Reaction does not fully come on in many instances for hours. After an amputation, or the extirpation of a tumor, the larger arteries bleed, and we secure them. We wait a reasonable time for reaction. The pulse, perhaps, indicates that it has resulted, and, anxious to place our patient in a condition of comfort, we, after again carefully searching for bleeding vessels, close the wound and apply our dressings. After the lapse of four or six hours we are summoned to our patient on account of bleeding. When we arrive we find that there has resulted a reactive excitement in the heart and arteries. The patient has lost a considerable amount of blood, and, what is not less mischievous, the wound is distended with coagulum, and great irritation caused thereby. Indeed the painful distension is one cause of the continuance of the hemorrhage.

To arrest the hemorrhage we are now oblidged to open the wound, turn out the coagulated blood, and secure the arteries. This is an operation not less painful than the primary one, and, on account of the condition of the patient, more productive of irritation. But the loss of blood thus incurred, and the irritation and renewed shock, are not the only evils resulting. Notwithstanding our efforts to cleanse the wound of blood, much will remain adherent, and will have penetrated the tissues. The exposure of the wound to air favors the putrefactive process in the blood retained, and, when suppuration results, the sanies produced enters the circulation, and ichorrhæmia is the consequence. Whether we term this pyæmia or ichorrhæmia, it can not be doubted that it far more frequently results where secondary hemorrhage occurs and the wound becomes stuffed with blood. The impairment of the vital powers, caused by the loss of blood, contributes to the same result.

I appeal to all surgeons whose observations have extended over the period of half a century passed, as mine have done, to say whether pyæmia has not been far more common as the fatal issue of surgical operations, than it was before chloroform was introduced? Has not this form of disease occupied far more of the attention of surgeons than it formerly did? Does not this frequent sequel of surgical operations at this time fill the minds of surgeons with dread? Does not the fact that far more has been written about pyæmia, since chloroform was introduced than ever before, give evidence of its more frequent and fatal occurrence?

There are other modes in which the use of powerful anæsthetics promote this pyæmic condition. The agent powerful enough to make such an impression upon the system as to make it insensible to the pain of a formidable operation, must very powerfully influence the functions of the nervous system, and morbidly modify them. The constitution of the blood must also be more or less modified.

But there are cases in which surgical manipulations are practiced in which incisions and the loss of blood are not involved—as, for instance, those employed in the treatment of dislocations and of strangulated hernia.

In the reduction of the hip and shoulder a double advantage is obtained by the use of chloroform. We not only obviate pain, but we overcome the rigidity and resistance of muscles which otherwise often render our efforts ineffectual. Here there is no danger of pyæmia, and the most formidable objection to the use of chloroform does not obtain.

The same may be said in regard to its employment in promoting the reduction of strangulated hernia. Here we may carry its influence to a greater extent than where the powers of life may be expected to be reduced by loss of blood.

I have recently reduced the femur in two cases, dislocated upward and backward on the ileum, employing chloroform, using only the force of my own hands. The process was by manipulation as taught and practiced more than half a century ago by my father, Prof. N. Smith. It consisted in flexing the thigh forcibly on the pelvis, carrying the knee toward the opposite hypochondrium, then strongly abducting the thigh, and suddenly extending both thigh and leg. The reduction was effected in a moment.

In employing chloroform to promote these reductions its effects must be carried far enough to cause complete relaxation. Carried to a certain degree—not complete—there is produced a spastic rigidity of the muscles which renders reduction far more difficult.

I should by no means keep out of view the great advantage derived from the use of chloroform in the execution of those operations—on the eye, for instance—which require that the patient should be completely passive and non-resistant. The most resolute can not refrain from a degree of struggle when an exceedingly sensitive organ is touched by an instrument. Here also the effect of chloroform must be complete or the effort of the partially insensible patient is more uncontrollable than ever. In all these cases, however, in which it must be carried thus far, the risk of fatal results is increased.

Nor should I neglect here to speak of the advantages derived from anæsthesia in cases of nervous and timid persons who would refuse surgical operations but for the confidence which they place in chloroform. Not only are their fears allayed, but their nervous systems are fortified against shock.—*Baltimore Medical Journal.*

